# 

**Published:** 1924-07

**Authors:** 


### THE Hospital and Health REVIEW

A JOURNAL FOR EVERYONE INTERESTED IN PUBLIC HEALTH, PREVENTIVE AND CURATIVE MEDICINE AND HOSPITAL AND INSTITUTIONAL WORK.

*Established 1886.*

### Published MONTHLY, Price 6d.

THE HOSPITAL AND HEALTH REVIEW includes within its sphere the various aspects of PUBLIC AND PERSONAL HEALTH, PREVENTIVE AND CURATIVE MEDICINE, HOSPITAL AND INSTITUTION WORK, AND THE GENERAL WELL-BEING OF THE PEOPLE. These subjects of truly vital concern are dealt with in plain, and as far as possible non-technical, language, so that they may be understood, and consequently of *service to everyone* interested in these matters.

Price 6d. of all Booksellers and Newsagents, or 7½d. post free of the Manager, "The Hospital and Health Review," 28/29 Southampton Street, Strand, London, W.C. 2.

### The Library of the

### Bristol Medico-Chirurgical Society.

*The following donations have been received since the publication of the List in January,* 1924.

*July,* 1924.

| | |
|---|---|
| Dr. W. Evans (1) .. .. .. .. | 1 volume. |
| Mr. Hey Groves (2) .. .. .. .. | 4 volumes. |
| Medical Research Council (3) .. .. .. | 2 ,, |
| Middlesex Hospital (4) .. .. .. .. | 1 volume. |
| Mr. A. B. Prowse (5) .. .. .. .. | 3 volumes. |
| The Surgeon-General, United States Army (6) | 1 volume. |

Unbound periodicals have been received from Dr. Carey Coombs and Mr. Hey Groves.

### THE ONE HUNDRED AND NINTH LIST OF BOOKS.

The figures in round brackets refer to the figures after the names of the donors. The books to which no such figures are attached have been received through the *Journal.*

| | | | |
|---|---|---|---|
| **Abraham, J. J.** .. | *Lectures on Gonorrhœa in Women* .. .. | | 1924 |
| **Allan, G. A.** .. | *Defects in Cardiac Rhythm in Relation to Cardiac Failure* .. .. .. .. | 2nd Ed. | 1924 |
| **Begg, A. C.** .. .. | *Insulin in General Practice* .. .. .. .. | | 1924 |
| **Bland-Sutton, Sir J.** | *Orations and Addresses* .. .. .. .. .. | | 1924 |
| **Brawn, H.** .. .. | *Local Anæsthesia* .. .. .. .. | 2nd Ed. | 1924 |
| *Cancer Research at the Middlesex Hospital* .. .. .. .. | | (4) | 1924 |
| *Catalogue (Index) of the Medical Library of the University of Aberdeen* | | (7) | 1923 |
| **Cripps, L. D.** .. | *The Application of the Air Force* .. .. | (3) | 1924 |
| **Crofton, W. M.** .. | *An Outline of Endocrinology* .. .. .. | | 1924 |
| **Cuff, H. E.** .. .. | *Practical Nursing* .. .. .. .. | 6th Ed. | 1924 |
| **Douglas and J. G. Priestley.** | *Human Physiology* .. .. .. .. | | 1924 |
| **Evans, W.** .. .. | *Diseases of the Breast* .. .. .. .. | (1) | 1923 |
| **Fairburn, J. S.** .. | *Gynæcology with Obstetrics* .. .. .. .. | | 1924 |
| **Gardiner, F.** .. .. | *Handbook of Skin Diseases* .. .. | 2nd Ed. | 1924 |

| | | | |
|---|---|---|---|
| **Girdlestone, G. R.** | *The Care and Cure of Cripple Children* .. .. | | 1924 |
| **Ham, B. B.** .. .. | *A Synoptic Chart of Skin Diseases* .. .. | | 1923 |
| **Jack, W. R.** .. .. | *Wheeler's Handbook of Medicine* .. | 7th Ed. | 1924 |
| **Joslin, E. P.** .. | *The Treatment of Diabetes Mellitus* | 3rd Ed. | 1924 |
| **Kidd, F.** .. .. | *Common Infections of the Female Urethra* .. | | 1924 |
| **MacLean, H.** .. | *Modern Methods of Glycosuria and Diabetes* | 2nd Ed. | 1924 |
| **Mummery, J. H.** .. | *The Microscopic and General Anatomy of the Teeth* .. .. .. .. .. .. .. | 2nd Ed. | 1924 |
| *Practitioner's Pocket Book, The* .. .. .. .. .. .. .. .. .. | | | 1924 |
| **Priestley and C. G. Douglas.** | *Human Physiology* .. .. .. .. | | 1924 |
| **Ross, T. A.** .. .. | *The Common Neuroses* .. .. .. .. .. | | 1923 |
| **Simpson, A. M.** .. | *Common Infections of the Female Urethra* .. | | 1924 |
| **Singer, D. W.** .. | *Ambroise Paré* .. .. .. .. .. .. .. .. | | 1924 |
| *Spas of Britain, The* | .. .. .. .. .. .. .. .. .. .. .. .. | | 1924 |
| **Stephenson, T.** .. | *Incompatibility in Prescriptions* .. .. .. | | 1924 |
| **Still, G. F.** .. .. | *Common Disorders and Diseases of Childhood* | 4th Ed. | 1924 |
| **Thomson, Sir S.** .. | *Tuberculosis of the Larynx* .. .. .. | (3) | 1924 |
| **Tredgold, A. F.** .. | *Mental Deficiency* .. .. .. .. .. | 4th Ed. | 1922 |
| **Turner, A. L.** .. | *Diseases of the Nose, Throat, and Ear* .. .. | | 1924 |
| **Varley, G. H.** .. | *Radium* .. .. .. .. .. .. .. .. .. .. | | 1924 |
| *Wheeler's Handbook of Medicine* | .. .. .. .. .. .. | 7th Ed. | 1924 |

### TRANSACTIONS, REPORTS, JOURNALS, etc.

| | | |
|---|---|---|
| American Association of Genito-Urinary Surgeons, Transactions of the .. .. .. .. .. .. .. .. .. .. .. | Vol. XVI. | 1923 |
| American Laryngological Association, Transactions of the | 3 Volumes, 1920, 1921, | 1922 |
| American Physicians' Association, Transactions of the | Vol. XXXVIII. | 1923 |
| Medical Society's Transactions, The .. .. .. .. | Vol. XLV. | 1923 |
| Medical Year Book, The .. .. .. .. .. .. .. .. .. .. .. | | 1924 |
| Ophthalmological Society, Transactions of the.. .. | Vol. XLIII. | 1923 |

### Local Medical Notes.

LONG FOX LECTURE.—Emeritus Professor Lloyd Morgan will deliver the Long Fox Lecture at 5 p.m. on July 23rd in the Physiological Theatre, University of Bristol. Subject: "The Conditional Response as a turning-point in Evolution."

### EXAMINATION RESULTS.

University of Bristol.—Students of the University have recently passed the following :—

M.B., Ch.B.—*Final Examination, Part I. (including Forensic Medicine and Toxicology) :* A. T. Binnie, A. S. Cox, B. V. F. Dawkins, C. H. Durnford, F. J. Farr, C. F. R. Killick, W. G. R. Morris, W. S. Ormiston, Y. de la Pasture, M. P. Posthuma, H. J. Satchwell. *Part I. only :* R. V. Cooke. *Forensic Medicine and Toxicology :* G. M. Minifie. *Part II., completing examination :* S. H. Blacker, G. R. Dunn, L. Dunn, E. B. Eedle, P. G. Evans, W. A. Gornall, J. L. Griffin, F. Langford, G. S. Mundy, J. R. Nicholson-Lailey, A. E. Sherwell.

D.B.S.—1*st Examination :* W. M. Evans.

L.D.S.—*Final Examination :* G. C. Brooks, A. W. Cawsey, G. C. Friend, H. F. D. Lane, B. F. Robinson.

D.P.H.—*Part I. :* J. Ledingham.

### APPOINTMENTS.

J. A. Nixon, C.M.G., M.D., F.R.C.P., has been appointed Professor of Medicine and Director of Clinical Medicine in the University of Bristol.

F. H. Edgeworth, M.A., M.D., D.Sc., has been appointed Consulting Physician to the Bristol Royal Infirmary.

Richard Clarke, O.B.E., M.B., M.R.C.P., has been appointed Physician to the Bristol Royal Infirmary.

A. T. Todd, O.B.E., M.B., Ch.B., M.R.C.P., has been appointed Assistant Physician to the Bristol Royal Infirmary.

Agnes Stuart, M.D., has been appointed Physician to the Maternity Hospital, Brunswick Square, Bristol.

Victoria Tryon, M.B., Ch.B., and Madge Golding, M.B., Ch.B., have been appointed Assistant Physicians to the Maternity Hospital, Brunswick Square, Bristol.

R. H. Hatcher, M.B., Ch.B., has been appointed Assistant Medical Officer to the Southmead Hospital, Bristol.

### *PUBLICATIONS RECEIVED.*

From Henry Frowde and Hodder & Stoughton, London:

*The Treatment of Fractures in General Practice.* By C. Max Page, D.S.O., M.S. (Lond.), F.R.C.S. and W. Rowley Bristow, M.B., M.S. (Lond.), F.R.C.S. 1924. First Edition. Price 12s. 6d. net.

From William Heinemann, London:

*Ear, Nose, and Throat Treatment in General Practice.* By Georges Portmann, M.D. Translated by R. Scott Stevenson, M.D. 1924. Price 10s. 6d. net.

*Organic Substances, Sera and Vaccines.* By D. W. Carmalt-Jones, M.D., F.R.C.P. 1924. Price 15s. net.

*Lectures on Gonorrhœa in Women and Children.* By J. Johnston Abraham, C.B.E., D.S.O., M.A. (Dub.), F.R.C.S. (Eng.). 1924. Price 7s. 6d. net.

*Orations and Addresses.* By Sir John Bland-Sutton. 1924. Price 10s. 6d. net.

From Henry Kimpton, London:

*The Treatment of Diabetes Mellitus.* By Elliott P. Joslin, M.D. (Harvard), M.A. (Yale). Third Edition. 1924. Price 36s. net.

From H. K. Lewis & Co. Ltd., London:

*Synopic Chest of Skin Diseases.* By Burnett Ham, M.D., D.P.H. 1923. Author of *A Handbook of Sanitary Law.* 1924. Price 12s. 6d. net.

*Practical Forensic Medicine.* By C. Graham Grant, V.D., L.R.C.P. Third Edition. 1924. Price 4s. 6d. net.

From Oxford Medical Publications, London:

*Common Infections of the Female Urethra and Cervix.* By Frank Kidd, M.A., M.Ch. (Cantab.), F.R.C.S. (Eng.), and A. Malcolm Simpson, B.A., M.B., D.P.H. (Cantab.). 1924. Price 7s. 6d. net.

*The Microscopic and General Anatomy of the Teeth.* By J. Howard Mummery, C.B.E., F.R.C.S., D.Sc. (Penn.), L.D.S. (Eng.). Second Edition. 1924. Price 25s.

*Gynæcology with Obstetrics.* By J. S. Fairbairn, M.A., P.M., B.Ch. (Oxon.), F.R.C.P. (Lond.), F.R.C.S. (Eng.). 1924. Price 25s. net.

*Human Physiology.* By C. G. Douglas, C.M.G., M.C., D.M., F.R.S. and J. G. Priestley, M.C., D.M., 1924. Price 12s. 6d. net.

*Radium: Its Therapeutic Uses in General Practice.* By G. H. Harvey, M.D. (Oxon.). 1924. Price 6s. net.

From Alexander Stenhouse, Glasgow:

*Defects in Cardiac Rhythm in Relation to Cardiac Failure.* By Geo. A. Allan, M.B., F.R.F.P.S.G. Second Edition. 1924. Price 1s. net.

From The University Press, London:

*The Diseases of the Breast.* By Willmott H. Evans, M.D., B.S., F.R.C.S. 1923. (Presented by the Author.)

From John Wright & Sons, Ltd., Bristol:

*Diseases of the Nose, Throat and Ear.* By A. Logan Turner, M.D., F.R.C.S. (Edin.). 1924. Price 20s. net.

*Epidemic Encephalitis.* By Arthur J. Hall, M.A., M.D. (Camb.), F.R.C.P. (Lond). 1924. Price 12s.

